# The 25(OH)D3, but Not 1,25(OH)2D3 Levels Are Elevated in IBD Patients Regardless of Vitamin D Supplementation and Do Not Associate with Pain Severity or Frequency

**DOI:** 10.3390/ph14030284

**Published:** 2021-03-22

**Authors:** Anna Zielińska, Aleksandra Sobolewska-Włodarczyk, Maria Wiśniewska-Jarosińska, Anita Gąsiorowska, Jakub Fichna, Maciej Sałaga

**Affiliations:** 1 Department of Biochemistry, Faculty of Medicine, Medical University of Lodz, Mazowiecka 6/8, 92-215 Lodz, Poland; ania.zielinska0122@gmail.com (A.Z.); jakub.fichna@umed.lodz.pl (J.F.); 2 Department of Gastroenterology, Faculty of Medicine, Medical University of Lodz, Plac Gen. Józefa Hallera 1, 90-647 Lodz, Poland; olasobolewska1@poczta.onet.pl (A.S.-W.); majkawj@bmp.net.pl (M.W.-J.); anita.gasiorowska@umed.lodz.pl (A.G.)

**Keywords:** vitamin D, inflammatory bowel diseases, Crohn’s disease, ulcerative colitis, supplementation, deficiency

## Abstract

Due to its immunomodulatory effect, vitamin D has been associated with clinical parameters and outcomes in inflammatory bowel diseases (IBDs) which are chronic conditions of the gastrointestinal tract. Upon synthesis or digestion, vitamin D is metabolized in the liver to form 25(OH)D3, the major circulating metabolite. Further renal hydroxylation generates 1,25(OH)2D3, the most potent metabolite. Our aim was to examine the association between vitamin D levels, and its supplementation and pain intensity in 39 IBD patients and 33 healthy individuals. 25(OH)D3 and 1,25(OH)2D3 serum levels were measured. Each subject filled out visual analog scale (VAS) and Laitinen’s pain assessment scales. Laboratory results were obtained, and disease activity was assessed. Linear regression was employed to investigate the correlation between 25(OH)D3, 1,25(OH)2D3 and pain intensity, clinical activity parameters, C-reactive protein, disease duration, and dietary habits. In IBD patients, 25(OH)D3 was increased, whereas 1,25(OH)2D3 was not. Vitamin D3 supplementation did not influence their levels. No correlation was found between pain scores, disease activity, inflammatory status, disease duration or dietary habits and both forms of vitamin D. Elevated 25(OH)D3 and normal 1,25(OH)D3 were found in IBD patients as compared to the controls. We discovered no effect from supplementation and no association between pain severity and vitamin D.

## 1. Introduction

Inflammatory bowel diseases (IBDs), including Crohn’s disease (CD) and ulcerative colitis (UC), are chronic, disabling disorders affecting mostly the gastrointestinal (GI) tract. IBD symptomatology is diverse and non-specific. General symptoms (fever, weakness, chronic diarrhea and weight loss) are accompanied by precipitating inflammation and ulceration throughout the GI tract [1,2]. Chronic abdominal pain is a frequent symptom of IBD, with up to 70% of the patients reporting it during the exacerbation, and more than 30% during clinical remission of the disease [2,3]. Pain is a cause of decreased quality of life in IBD patients, especially since the relapsing course of the disease causes intermittent exacerbations of pain [2,4]. It can occur secondary to acute inflammation, adhesions, bowel obstruction or strictures, bowel dysmotility, intestinal fistulas and abscess formation, but also because of extraintestinal symptoms of IBD, such as arthritis, skin lesions, bone diseases or oral aphthous lesions [2,3]. Pain in IBD is also influenced by commonly experienced fatigue, psychological factors, prior surgeries and relapses or disease activity [3]. Aside from the abovementioned, pain may be caused by visceral hypersensitivity, changes in sensory pathways during active inflammation, bacterial overgrowth or concomitant irritable bowel syndrome [3,5]. Importantly, skeletal and muscle pain have been associated with vitamin D deficiency, another very common ailment in IBD patients [3,6], although no such association has been established in IBD so far.

Vitamin D level is one of the environmental factors that may influence predisposition to IBD or its course [7]. It has been suspected to play an important immunologic role in IBD, particularly due to its anti-inflammatory effects [1,7]. It regulates gut epithelial integrity, microbiota, immune barrier function and growth and function of T cells, mechanisms essential in the prevention and improvement of the disease’s symptoms [1,6]. Vitamin D deficiency in IBD is much more prevalent than in the general population, and higher in CD than in UC [1]. It is due to small bowel inflammation, causing malabsorption of nutrients, frequent ileal resection affecting bile salt resorption, frequent defecations, dietary restrictions, sun avoidance during immune-suppressive therapies and spending more time indoors due to disease exacerbations [1,8,9]. The evidence that vitamin D supplementation cures or prevents IBD is uncertain. Despite the growing popularity of vitamin D supplements, inflammatory diseases are still on the rise [10]. Currently, there are no specific guidelines advising appropriate supplemental doses [6].

25-hydroxyvitamin D3 (25(OH)D3) is the inactive, but main circulating form of vitamin D [10,11]. In the renal tubular epithelium, it is hydroxylated to a physiologically active form and is the most potent steroid hormone in the human body: 1,25-dihydroxyvitamin D3 (1,25(OH)2D3) [9,10,11,12]. There is a notion that levels of serum 25(OH)D3 are accurate reflections of vitamin D status. However, in autoimmune diseases, 1,25(OH)2D3 levels may be elevated, while 25(OH)D3 is deficient or normal, so its measurement may not provide enough data on vitamin D status [10].

The aim of our study was to determine if there is an association between the vitamin D forms’ levels and the severity of pain experienced by IBD patients. Moreover, we took a closer look at vitamin D levels in connection with vitamin D supplementation with over-the-counter (OTC) drugs as well as disease activity, the severity of inflammation measured with C-reactive protein (CRP), and disease duration.

## 2. Results

### 2.1. Study Group

The demographics, laboratory outcomes and disease characteristics of the study population are summarized in Table 1. A total of 39 IBD patients and 33 healthy controls were enrolled in the study. In the IBD group, the majority were male with no significant differences in age between the CD and UC patients. Their questionnaires and medical charts were reviewed, and their serum 25(OH)D3 and 1,25(OH)2D3 levels were measured and then compared with the control group’s results. 

### 2.2. 25OH D3 and 1,25(OH)2D3 Levels

**Figure 1:**
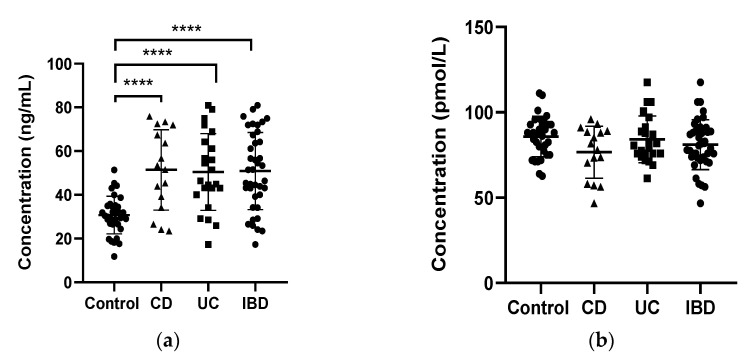
Serum concentrations of 25-hydroxy Vitamin D (25(OH) Vitamin D) (**a**) and 1,25-hydroxy Vitamin D (1,25(OH)2 Vitamin D) (**b**) in healthy subjects (control), in patients with Crohn’s disease (CD) and ulcerative colitis (UC) and the whole test IBD patient group. **** *p* < 0.0001, the difference between CD and UC was not statistically significant.

We discovered that serum 25(OH)D3 levels were significantly higher in the CD, UC and IBD group in comparison to the healthy volunteers (51.43 ± 4.59, 50.44 ± 3.66 and 50.85± 2.83 vs. 30.37 ± 1.51 in CD, UC, IBD and control, respectively; *p* < 0.0001). The differences between CD patients and UC patients lacked statistical significance. On the other hand, serum 1,25(OH)2D3 was not different in IBD patients compared to healthy volunteers (76.64 ± 3.80, 84.13 ± 2.86 and 81.06 ± 2.34 vs. 85.84 ± 2.10 in CD, UC, and IBD patients and the control, respectively; *p* < 0.0001) (Figure 1). 

### 2.3. Vitamin D3 Supplementation

**Figure 2:**
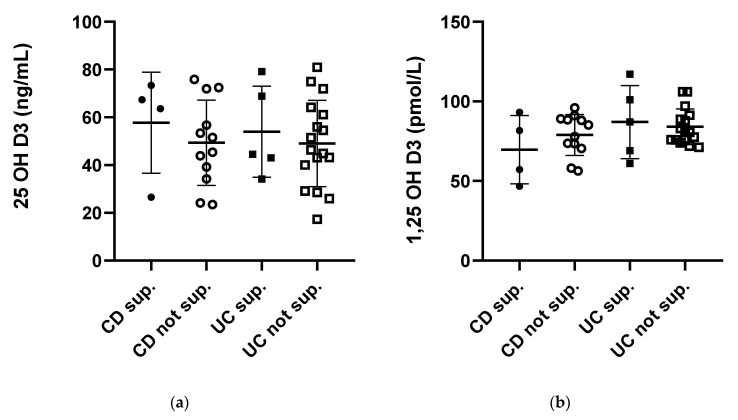
Serum concentrations of 25-hydroxy Vitamin D (25(OH) Vitamin D) (**a**) and 1,25-hydroxy Vitamin D (1,25(OH)2 Vitamin D) (**b**) in Crohn’s disease (CD) and ulcerative colitis (UC) patients supplementing (sup.) or not supplementing (not sup.) vitamin D with over-the-counter (OTC) drugs.

Vitamin D3 supplementation (1500–4000 I.U./daily) with commercially available over-the-counter drugs did not influence serum levels of 25(OH)D3 (in CD: 49.34 ± 5.16 vs. 57.71 ± 10.59 without supplementation vs. supplemented; in UC: 49.04 ± 4.38 vs. 53.94 ± 8.54 without supplementation vs. supplemented) and 1,25(OH)2D3 (in CD: 78.96 ± 3.72 vs. 69.70 ± 10.73 without supplementation vs. supplemented; in UC: 84.03 ± 2.19 vs. 87.00 ± 10.24 without supplementation vs. supplemented). Data on the relation between vitamin D supplementation and serum 25(OH)D3 and 1,25 (OH)2D3 levels are shown in Figure 2. The number of participants who consumed vitamin D supplements in each study group was low; however, they reported daily supplementary intake of vitamin D (Table 1).

### 2.4. Disease Activity

**Figure 3:**
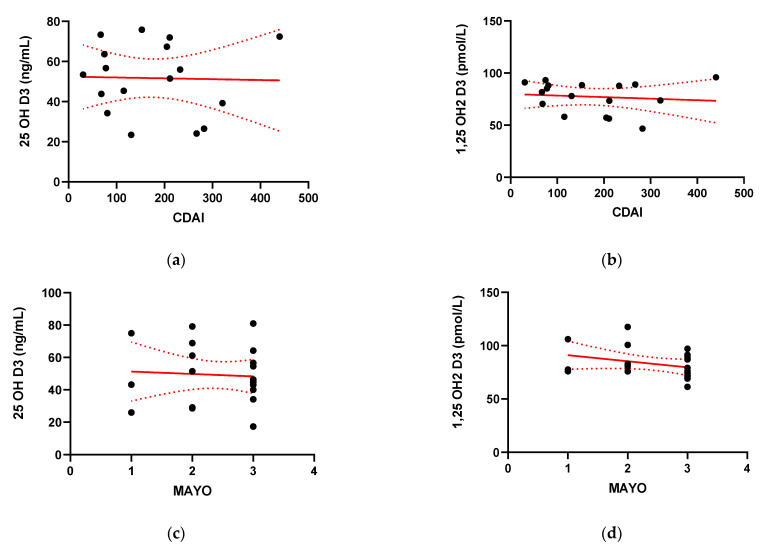
Correlation between serum concentration of 25-hydroxy Vitamin D (25(OH) Vitamin D) (**a**) and 1,25-hydroxy Vitamin D (1,25(OH)2 Vitamin D) (**b**) and the value of the Crohn’s Disease Activity Index for Crohn’s disease (CD) patients (**c**); and the value of Mayo score (c and d respectively) for ulcerative colitis (UC) patients (**d**).

The majority of the CD patients presented with a mildly to moderately active disease, with a mean CDAI of 180.47 ± 110.32. In UC patients, the course of the disease was more severe and most of the UC patients were scored 3 in the endoscopic Mayo assessment. The mean endoscopic Mayo score was 2.43 ± 0.75. CDAI and Mayo scores were analyzed for association with serum 25(OH)D3 and 1,25(OH)2D3 levels. There were no significant correlations between 25(OH)D3 or 1,25(OH)2D3 and the CDAI or Mayo score (Figure 3). 

### 2.5. Inflammatory Status, Duration of the Disease and Pain

Other individual variables were next analyzed for correlations with serum 25(OH)D3 or 1,25(OH)2D3 as described in the methods section. Since there were no significant differences in 25(OH)D3 and 1,25(OH)2D3 levels between CD and UC patients, these groups were combined for analyses.

**Figure 4:**
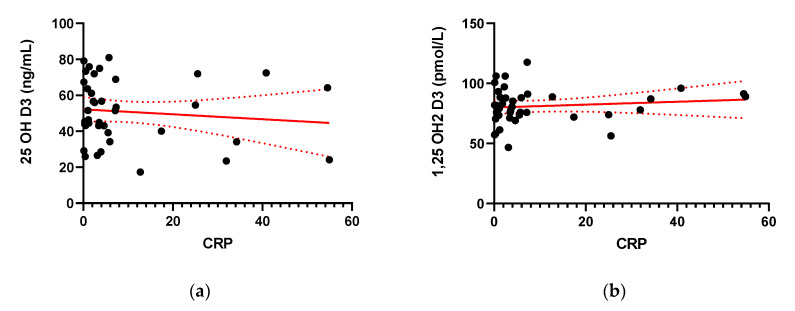
Correlation between serum concentration of 25-hydroxy Vitamin D (25(OH) Vitamin D) (**a**) and 1,25-hydroxy Vitamin D (1,25(OH)2 Vitamin D) (**b**) and the levels of C-reactive protein (CRP) in IBD patients.

The mean CRP in IBD patients in our study equaled 9.71 ± 14.76. The CRP levels did not correlate positively with serum 25(OH)D3 or 1,25(OH)2D3 levels (Figure 4).

**Figure 5:**
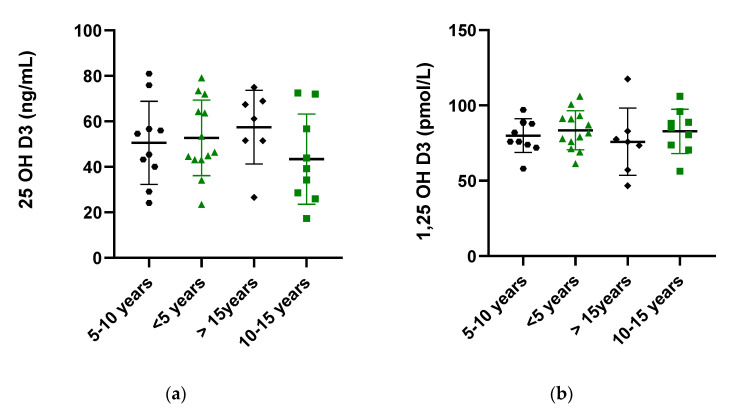
Serum concentration of 25-hydroxy Vitamin D (25(OH) Vitamin D) (**a**) and 1,25-hydroxy Vitamin D (1,25(OH)2 Vitamin D) (**b**) in IBD patients with respect to disease duration.

The duration of the disease also did not correlate significantly with the levels of 25(OH)D3 or 1,25(OH)2D3 (Figure 5). 

**Figure 6:**
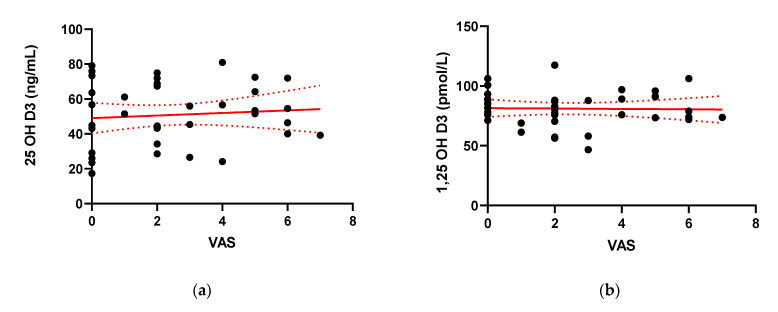

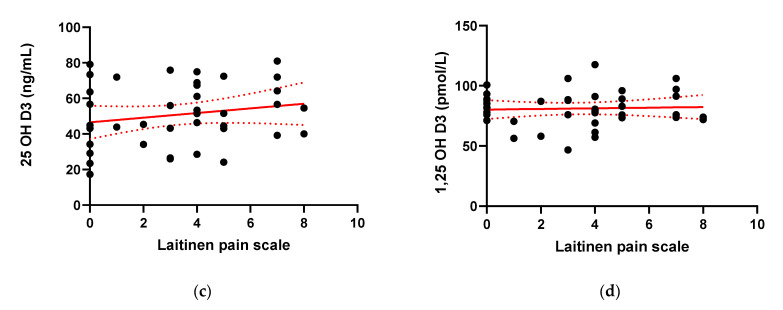
Correlation between serum concentration of 25-hydroxy Vitamin D (25(OH) Vitamin D) (**a**) and 1,25-hydroxy Vitamin D (1,25(OH)2 Vitamin D) (**b**) and the value of the visual analog scale (VAS) for quantification of disease-related pain (**c**); and the value of the modified Laitinen Pain Scale (c and d respectively) in IBD patients (**d**).

The pain experienced and subjectively assessed by IBD patients in the VAS pain scale and Laitinen’s scale did not show statistically important correlation with both serum vitamin D forms [Figure 6]. The presence of pain was reported by 76.9% of patients (30/39). The mean scores for pain severity were similar for the CD and UC groups. For simplification of the statistical analysis, Laitinen’s scale points have been added. The scale and mean results of the questionnaire are presented in Table 2.

No correlation was found between the mean platelet volume, extraintestinal symptoms and body mass index. Dietary habits of IBD patients also did not influence the quantity of serum vitamin D (data presented in Supplementary Materials). Other initially investigated variables, comprising white blood cells (WBC), blood platelets (PLT), red blood cells (RBC), hematocrit (Hct) and hemoglobin, did not influence vitamin D serum levels or revealed statistical significance, and therefore were not included.

## 3. Discussion

We found increased 25(OH)D3 levels in IBD patients, and no difference in 1,25(OH)2D3 levels compared to healthy volunteers. Supplementation of vitamin D in enrolled IBD patients did not cause an increase in either metabolites. We did not find any association between vitamin D levels and disease activity, inflammatory status and pain severity or frequency.

Vitamin D supplementation is generally recommended for IBD, especially for the management of the bone disease. Not all studies, however, have recognized vitamin D deficiency in IBD patients or the benefits of its intake [11,12,13]. For instance, Alkhouri et al. reported high vitamin D and zinc deficiency in both, 61 IBD patients and 61 sex and matched controls (62 and 75%), with no correlation to the disease [14]. Steroid usage is often suspected to cause a reduction in bone mineral density (BMD) and vitamin D deficiency in IBD as they increase 24-hydroxylase activity which catabolizes 1,25(OH)2D3 and 25(OH)D3 into their inactive metabolite: calcitroic acid. This was contradicted in a study by Lamb et al., in which bone mineral density was decreased even before steroid initiation [15]. Moreover, no correlation between increased incidence of UC and low sun exposure is reported, except for one between sunlight and CD [16]. In our study, blood samples were collected in a three-month period (between February and April); therefore we did not analyze the results separately by season for each IBD group. On the other hand, in a study performed by Ananthakrishnan et al., an increased IBD-related surgery risk was related to low serum 25(OH)D3 levels. CD patients, whose levels were normalized, were less susceptible to surgery than CD patients with continuously low serum levels of 25(OH)D3. They also presented a normalized CRP level. In UC patients, however, no correlation was found, which might be due to a stronger interaction of vitamin D in CD [17]. In another study performed by this group in 2012 on 72,719 women to find a risk of developing CD and UC, a group presenting with the highest predicted plasma vitamin D level had a much lower (40% reduced) risk of developing CD, but no reduced risk of UC [18]. Contrarily, Nielsen et al., in a prospective cohort study of 120,013 individuals, described higher 25(OH)D3 levels as being associated with an increased risk of UC, but not of CD. However, genetically high plasma 25(OH)D levels (the combined 25(OH)D increasing allele score) were not associated with CD or UC. Thus, those results did not support a major role for vitamin D deficiency in the development of CD or UC [19].

The current method of defining vitamin D is flawed, as the level of 25(OH)D3 is not always reflective of the 1,25(OH)2D3 level. The latter is often increased when the former is normal or decreased, which could be a sign of changed vitamin D endocrine function. Our study showed higher serum 25(OH)D3 levels in IBD patients than in healthy controls, and comparable 1,25(OH)2D3 levels. This is contradictory to most studies that reported low 25(OH)D3 levels as a consequence of chronic inflammation or bacterial etiology [10]. Moreover, increased inflammation has been associated with enhanced extra-renal conversion of 25(OH)D3 to 1,25(OH)2D3 to support the anti-inflammatory immune response of the GI tract [1]. This extra-renal production of 1,25(OH)2D3 contributes to the depletion and low levels of 25(OH)D3. Because extra-renal 1,25(OH)2D3 production is dependent on 25(OH)D3 availability, the supplementation of vitamin D to increase 25(OH)D3 levels boosts extra-renal 1,25(OH)2D3 production [10]. Our results may imply a disruption in 1,25(OH)2D3 synthesis or a decreased response to parathyroid hormone, a dominant stimulant of 1,25(OH)2D3 synthesis in IBD patients. In the literature, inflammation is associated with lower PTH and 1,25(OH)2D3 concentrations [20]. In some studies, serum levels of 1,25(OH)2D3 were lower in IBD patients than in healthy patients due to improved bone mineral density (BMD) after remission of IBD, making 1,25(OH)2D3 normal [21]. 

The measurement of 1,25(OH)2D3 is rare, but this metabolite correlates inversely with BMD; it stimulates calcium absorption from the intestines, reabsorption in the kidneys and resorption in the bones, increasing bone loss and IBD severity [1,22]. Vanderschueren et al. described a combination of high 1,25(OH)2D3 and low 25(OH)D3 as a predictor of the poorest bone health [23]. Our patients presented opposite results. 

Our study did not reveal any significant difference in 25(OH)D3 or 1,25(OH)2D3 levels between the CD and UC group. Abreu et al., on the contrary, demonstrated higher 1,25(OH)2D3 statuses in CD patients compared to UC patients [12]. The Nurses’ Health Study proved that 25(OH)D3 protected patients from developing CD, but not UC [22]. In two prospective studies, CD patients with 25(OH)D3 levels lower than 30 ng/mL required more hospitalizations and surgeries than CD patients with higher 25(OH)D3 entry levels [21]. One of these studies included UC patients, in which the level of <30 ng/mL resulted in treatment intensification and increased morbidity in a five-year follow up. The same result was obtained for CD patients [24]. 

In the current study, vitamin D supplementation influenced neither 25(OH)D3, nor 1,25(OH)2D3 levels of IBD patients. Most of the studies conducted to date reported that in IBD, oral supplementation may lead to vitamin D status improvements and benefits from the normalization of 25(OH)D3 levels [1]. In a study by Grunbaum et al., patients supplementing vitamin D supplements had higher levels than those who depended only on dietary intake. Yet, the levels of vitamin D still did not reach the desired values; about 50% of the patients and controls took supplements, but almost 60% did not achieve replete levels and supplementation was described as “disappointing”. An increase was noted in the patients’ group, but it was not statistically significant [25]. Furthermore, Suibhne et al. reported common vitamin D deficiency in CD patients in clinical remission, even though 40% of them were regularly supplementing vitamin D (200–400 IU a day) [26]. 

Many clinical trials analyzed the role of vitamin D3 supplementation in IBD activity. In a study by Narula et al., 34 CD patients in remission received 1000 or 10.000 IU a day for 12 months. In this randomized double-blind study, the intention-to-treat analysis showed no significant differences between the two groups and the per protocol analysis showed a decreased risk of relapse after 12 months in the high-dose group [27]. In a double-blind placebo-controlled trial by Palmer et al., 94 CD remitted patients received 1200 IU of vitamin D a day or a placebo for one year. Both groups also supplemented 1200 mg of calcium a day. The treatment insignificantly reduced CDAI [28]. In another randomized placebo-controlled study on 108 CD patients in remission, individuals receiving 1200 IU a day had increased 25(OH)D3 levels in comparison to the placebo group, as well as a reduced risk of relapse (29 to 13% in one year); the results, however, were not statistically significant [29]. Yang et al. in a small open label pilot study evaluated 18 moderate CD patients and their vitamin D status, quality of life and clinical disease activity. They supplemented vitamin D3 for 24 weeks, starting from 1000 IU a day. The dose was increased by 1000 IU every two weeks until the patients were taking 5000 IU a day or had serum 25(OH)D3 level >40 ng/mL. After the intervention, 78% of the patients presented CDAI scores below 150 and a decrease in CDAI score of 70 points or more. Moreover, their 25(OH)D3 levels and quality of life increased significantly [30]. Higher 25(OH)D3 levels were found to be associated with greater odds of remission with anti-TNF- α agents (adalimumab and infliximab) among patients with IBD in a study by Winter et al. Patients with normal vitamin D levels at the time of anti-TNF-α medication initiation had 2.64 increased odds of remission at three months compared to patients with low vitamin D levels. These findings suggest that vitamin D levels may influence the initial response to anti-TNF-α drugs and that low vitamin D levels could decrease the odds of remission [31].

There is strong evidence showing significantly lower 25(OH)D levels correlating with higher CDAI scores. Mildly diseased CD patients have vitamin D levels similar to the controls, while moderate to severe CD cases show significantly lower vitamin D levels [32].

In UC patients, a similar connection can be found. In a cross-sectional study, vitamin D deficient, sufficient and insufficient groups were compared. The larger number of UC patients with a clinically active disease described by a six-point partial Mayo score were in the vitamin D deficient group, compared to the vitamin sufficient group. There continued to be a trend towards more active diseases with decreasing vitamin D levels [33]. In our study, we did not find statistical correlation between IBD activity and vitamin D levels. This is in accordance with a report by Hassan et al., who also found no association between serum vitamin D levels and disease activity [34]. 

Vitamin D can also be connected with inflammatory markers of colitis: CRP and fecal calprotectin. We took CRP levels into consideration and found no significance when correlating it with 25(OH)D3 and 1,25(OH)2D3 levels. A Korean study by Jun et al., concordantly to our research, established no association between 25(OH)D3 levels, CRP and partial Mayo scores in UC. In CD, a negative correlation was found between 25(OH)D3 and CRP levels, but administration of vitamin D did not improve the CRP level and disease indexes [35]. Contrarily, Garg et al. described a significant increase in vitamin D levels and an associated significant CRP reduction in active UC, inactive UC patients and non-UC probands. Nonetheless, the dose of supplemented cholecalciferol was rather high: 40.000 a week [36]. In another trial, 90 UC patients in remission were randomized to receive 300.000 IU intramuscular vitamin D or 1 mL normal saline as a placebo. Before intervention and 90 days after intervention, serum levels of 25(OH)D3, PTH, calcium, ESR, and CRP were measured, and a significant decrease in CRP in the vitamin D group was reported [37].

Our study aimed at correlating disease duration with vitamin D levels and found that there is no correlation between the two. There is a limited body of literature concerning this association. A Japanese study by Tajika et al. found a relationship between vitamin D level and disease duration, CRP and CDAI, even though the CD patients did not present vitamin D levels lower than the control group. Moreover, it was also suggested that the duration of the disease and CDAI score can forecast the occurrence of vitamin D deficiency and that 25(OH)D3 levels should be assessed in patients who have had CD for a long time (>15 years) [38]. Suibhne et al. reported lower vitamin 25(OH)D levels to be associated with longstanding disease duration and smoking [26]. In a retrospective cohort study, Ulitsky et al. included 403 CD patients with a mean disease duration of 15.5 years and 101 UC patients with a duration of 10.9 years. Of these patients, 49.8% were vitamin D deficient, with 10.9% having severe deficiency. In this study, vitamin D deficiency was associated with older age and older age at diagnosis, and also with increased CDAI [39]. The temporal causation of vitamin D deficiency and disease onset has not yet been endorsed. Subclinical disease can exist prior to a correct diagnosis. In children, for example, a change in growth pace can serve as a disease predictor and has been found to precede the formal diagnosis by 3–92 months [40,41].

In this study, neither pain severity, nor its frequency or influence on motor activity were significantly correlated with 25(OH)D3 or 1,25(OH)2D3 levels in IBD patients. There is only one study that investigated the association between vitamin D deficiency and pain severity in IBD and it is by Frigstad et al. This group also found no significant association between vitamin D level and pain severity. Pain severity has been, however, linked with increased disease activity in both UC and CD, but not with inflammatory markers, namely CRP and fecal calprotectin [3], which was not substantiated in our study. Previously, deficient vitamin D has been found in musculoskeletal diseases, such as osteomalacia, musculoskeletal pains and headaches [2,42]. In IBD, low vitamin D levels can be found during flares of the disease when the pain experience is increased due to aggravated symptoms and elevated visceral sensitivity [2,43]. Moreover, vitamin D reduces intestinal inflammation and inhibits prostaglandin E, which is a known inflammatory pain mediator [3,42]. Yet, inflammation is not always present when abdominal pain is reported, which was described in a study on abdominal pain in UC. In the absence of ongoing inflammation management, strategies should include interventions that target affective spectrum disorders and functional bowel disorders [44]. 

## 4. Materials and Methods

Ethical considerations: The study was conducted in accordance with the ethical principles of the 1975 Declaration of Helsinki and the study protocol was approved by the Committee of Bioethics of Medical University of Lodz, Poland (RNN/621/14/KB, 15 July, 2014). 

Patients: This prospective observational cohort study was conducted in 39 IBD patients and 33 controls of Caucasian origin; 51.4% were male. The median age was 37 years (ranged 18–79 years). Subjects from the study group were patients qualified for biological treatment with infliximab, adalimumab and vedolizumab at the Department of Gastroenterology, Medical University of Lodz, Poland. Only adult patients with IBD were enrolled (CD nL’17 and UC nL’22). 

The control group consisted of healthy subjects (the staff of the Department of Biochemistry, Medical University of Lodz, Poland, who voluntarily participated in the study) and was homogenous to the study group in terms of age, sex, and body mass index. 

Inclusion criteria: The inclusion criteria for the study groups were based on the proper diagnosis according to clinical, radiological, endoscopic and histological criteria recommended by the European Crohn’s and Colitis Organization (ECCO). 

Patients with a history of cardiovascular disease, pulmonary and kidney disease, allergy, diabetes, lichen planus, psoriasis, atopic dermatitis and other autoimmune skin lesions and those treated with anti-inflammatory drugs (except azathioprine and corticosteroids), antioxidants or statins were excluded from the study.

25(OH)D3 and 1,25(OH)2D3 serum quantification: After the participants from both groups gave written consent, 5 mL blood samples were collected into standardized serum tubes. The serum was immediately separated and quickly frozen at –80 °C and stored until use. In the IBD group, the sample was obtained in addition to blood specimens collected routinely on the day of the infusion of anti-tumor necrosis factor-α (anti-TNF- α) agent (for standard blood work and clinical evaluation).

The total serum concentrations of 25(OH)D3 and 1,25(OH)2D3 were determined by the quantitative enzyme-linked immunosorbent assay (ELISA), using commercially available kits (OriGene, USA and Cusabio, USA respectively). Absorbances were read in a microplate reader at 450 nm (MicroPlate Reader; Bio-Rad, USA). For each detection, calibration blank tests have been taken into account. Each determination was carried out in duplicate in accordance with the manufacturers’ instructions. All steps of the ELISA test were conducted at room temperature according to the assays’ procedures. 

The serum for the determination of 25(OH)D3 was not diluted, whereas the dilution of the serum for quantification of 1,25(OH)2D3 equaled 1:10. 

The serum concentrations of 25(OH)D3 and 1,25(OH)2D3 were determined by calculating the mean absorbance values of reference standards, controls and patient samples and then using them to determine the corresponding concentration from the standard curve. In healthy persons, levels equal 25 to 40 ng/mL (62.4 to 99.8 nmol/L) for 25(OH)D3 and 20 to 45 pg/mL (48 to 108 pmol/L) for 1,25(OH)2D3 [45].

Demographic and clinical data: Each subject filled out a dietary, vitamin D supplementation and pain questionnaire (described below). Moreover, IBD patients were asked questions concerning the duration of the disease, extraintestinal symptoms and general wellbeing. The demographic data, including age, gender, body mass index, duration of the disease, the patient’s age at diagnosis and previous biological treatments, data concerning alcohol and drug consumption and smoking history were collected from the patients and analyzed. 

The laboratory evaluation of IBD patients included measurements of white blood cells (WBC), red blood cells (RBC), hemoglobin (Hgb), hematocrit (Htc), blood platelets (PLT), mean platelet volume (MPV) and C-reactive protein (CRP), determined using automatic devices. Measurements were performed on the day of the infusion of the anti-TNF agent (Table 1).

Assessment of disease activity: The disease activities of enrolled patients were assessed by abscesses or persistent symptoms despite intensive treatment was defined as severe CD using validated scales including the Crohn’s Disease Activity Index (CDAI) for CD and the endoscopic Mayo Score for UC, respectively [46]. According to the European Crohn’s and Colitis Organization (ECCO), the second European evidence-based consensus on the diagnosis and management of CD, CDAI <150 was defined as remission; CDAI 150–220 with no features of obstruction, fever, dehydration, abdominal mass or tenderness was defined as mild CD; CDAI 220–450 or intermittent vomiting, or weight loss >10% or ineffective treatment for mild disease, or tender mass with no overt obstruction was defined as moderate CD; CDAI <450 or cachexia (body mass index (BMI) <18 kg/m2), or evidence of obstruction or abscess or persistent symptoms despite intensive treatment was defined as severe CD.

For UC, clinical remission was defined as a Mayo score of 0; mild UC was defined as Mayo Score of 1; moderate UC was defined as a Mayo score of 2; severe UC was defined as a Mayo score of 3 [47].

Assessment of pain experienced by the patient: All the patients were asked to fill out two pain questionnaires. For pain assessment, two scales were used: a visual analog scale (VAS), which is a validated, subjective measure for acute and chronic pain [48]. Scores were recorded by making a handwritten mark on a 10 cm line that represents a continuum between “no pain” at 0 and “worst pain” at 10 and Laitinen’s pain scale, which allows for measuring pain intensity and simultaneously assessing other factors accompanying pain, namely frequency of pain occurrence, taking pain killers and limited motor activity (Table 2) [49].

Statistical analysis: The data were analyzed using the GraphPad Prism 8.0.1. (GraphPad Software, United States). Continuous demographic and biochemical data are presented as means ± standard deviation (SD), and demographic categorical data are described with absolute frequencies and percentages. An analysis of variance (one-way ANOVA with repeated measures) was used to calculate the differences. Shapiro–Wilk’s W test was used to test the distribution of the variables. Comparisons between groups were performed using the unpaired t-test and F test to compare variances. A value of *p* < 0.05 was considered statistically significant.

## 5. Conclusions

In our study, elevated 25(OH)D3 levels were associated with IBD, where the level of 1,25(OH)2D3 was similar to that of the healthy controls. This may imply a disruption in 1,25(OH)2D3 synthesis or a decreased response to parathyroid hormone, a dominant stimulant of 1,25(OH)2D3 synthesis in IBD patients. No reaction to vitamin D supplementation in the enrolled IBD patients was observed. This unexpected result may suggest diminished absorption or short longevity of ingested supplements. We did not find any association between vitamin D levels and pain severity or frequency. Further interventional studies are warranted to prove this hypothesis. 

A limitation of our study was that it was performed on a small group of IBD patients. Further studies are warranted to confirm our observations, especially since they are contradictive to several previous studies. The major strength was that the study and control groups were homogenous and the study was multifactorial. 

## Figures and Tables

**Table 1:** Socio-demographic and clinical data of inflammatory bowel disease (IBD) patients and healthy controls.

	IBD Patients	Control Group
**Subjects, n (%)**	39	33
	CD 17 (43.6%)	
	UC 22 (56.4%)	
**Sex**		
**Men, n (%)**	22 (56.4%)	15 (45.5%)
**Women, n (%)**	17 (43.6%)	18 (54.5%)
**Age, year**	35.74 ± 13.61	38.73 ± 17.52
**BMI, kg/m^2^**	32.21 ± 4.88	24.45 ± 4.05
**Duration of disease, n (%)**		
**<5 years**	13 (33.3%)	
**5–10 years**	10 (25.6%)	
**10–15 years**	9 (23.1%)	
**>15 years**	7 (19%)	
**Resection, n (%)**	6 (15.4%)	
**Colostomy, n (%)**	2 (5.1%)	
**Extraintestinal symptoms, n (%)**	23 (59.1%)	
**Use of painkillers, n (%)**	13 (33.3%)	4 (12.1%)
**NSAIDs**	12 (30.8%)	
**Opioids-Tramadol**	1 (2.6%)	
**Biological treatment, n (%)**		
**IFX**	33 (84.6%)	
**ADA**	2 (5.1%)	
**VDZ**	4 (10.3%)	
**CDAI, points**	180.47 ± 110.32	
**MAYO, points**	2.43 ± 0.75	
**Vitamin D3 supplementation, n (%)**		
**None**	30 (76.9%)	32 (97.0%)
**1500 IU**	3 (7.7%)	
**2000 IU**	5 (12.8%)	
**4000 IU**	1 (2.6%)	1 (3.0%)
**Mean 25(OH)D3, ng/mL**	50.85 ± 17.68	30.73 ± 8.65
**Mean 1,25(OH)2D3, pmol/L**	81.05 ± 14.64	85.84 ± 2.10
**White blood cell count, ×10^3^/µL**	8.14 ± 4.08	
**Red blood cell count, ×10^6^/µL**	4.57 ± 0.54	
**Hemoglobin, g/dL**	12.91 ± 3.01	
**Hematocrit, %**	39.02 ± 5.31	
**Platelet count, ×10^3^/µL**	358.08 ± 154.76	
**Mean platelet volume, fL**	9.91 ± 1.01	
**CRP, mg/dL**	9.71 ± 14.74	

**Abbreviations:** ADA: Adalimumab; BMI: Body mass index; CD: Crohn’s disease; CDAI: Crohn’s Disease Activity Index; CRP: C-reactive protein; IFX: Infliximab; NSAID’s: Non steroid anti-inflammatory drugs; UC: ulcerative colitis; VDZ: Vedolizumab.

**Table 2:** Laitinen’s pain scale results in enrolled IBD patients.

Factor	Subjective Evaluation	Points	n, %
**Pain Intensity**	Without pain	0	13 (33.3%)
	Mild	1	18 (46.2%)
	Strong	2	8 (20.5%)
	Very strong	3	0
	Not sustainable	4	0
**PAIN FREQUENCY**	Does not occur	0	12 (30.8%)
	Periodical	1	18 (46.2%)
	Frequent	2	8 (20.5%)
	Very frequent	3	0
	Continuous	4	1 (2.6%)
**PAINKILLERS’ INTAKE**	Without medication	0	13 (33.3%)
	Periodically	1	24 (61.5%)
	Permanently- small doses	2	2 (5.1%)
	Permanently- big doses	3	0
	Permanently- very big doses	4	0
**MOTOR ACTIVITY LIMITATION**	None	0	18 (46.2%)
	Partial	1	12 (30.8%)
	Demanding partial help/making work difficult	2	8 (20.5%)
	Demanding partial help/making work impossible	3	0
	Demanding full help/preventing self sufficiency	4	1 (2.6%)

## Data Availability

Not applicable.

## Supplementary Materials

The following are available online at https://www.mdpi.com/1424-8247/14/3/284/s1, **Figure S1.** Serum concentration of 25-hydroxy Vitamin D (25(OH) Vitamin D) (a) and 1,25-hydroxy Vitamin D (1,25(OH)2 Vitamin D) (b) in IBD patients applying protein and carbohydrate- rich diet or balanced diet. **Figure S2.** Correlation between serum concentration of 25-hydroxy Vitamin D (25(OH) Vitamin D) (a) and 1,25-hydroxy Vitamin D (1,25(OH)2 Vitamin D) (b) and the values of body mass index (BMI). **Figure S3.** Correlation between serum concentration of 25-hydroxy Vitamin D (25(OH) Vitamin D) (a) and 1,25-hydroxy Vitamin D (1,25(OH)2 Vitamin D) (b) and the values of mean platelet volume (MPV) in IBD patients. **Figure S4.** Serum concentration of 25-hydroxy Vitamin D (25(OH) Vitamin D) (a) and 1,25-hydroxy Vitamin D (1,25(OH)2 Vitamin D) (b) in IBD patients with and without extraintestinal symptoms.
