# Supplementary material for: The 25(OH)D3, but Not 1,25(OH)2D3 Levels Are Elevated in IBD Patients Regardless of Vitamin D Supplementation and Do Not Associate with Pain Severity or Frequency
Source: Pharmaceuticals (Basel). 2021 Mar 22;14(3):284. doi: 10.3390/ph14030284 (PMC8004142; doi:10.3390/ph14030284)
Supplement: Supplementary file 1 [file pharmaceuticals-14-00284-s001.pdf]

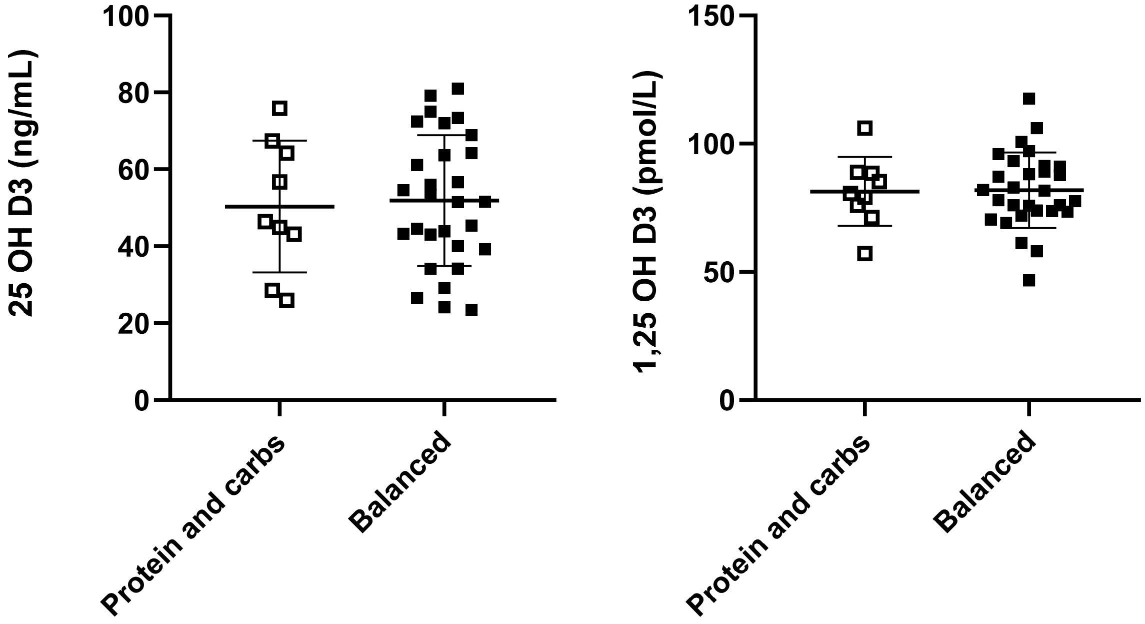

(**a**) (**b**)

Figure S1. Serum concentration of 25-hydroxy Vitamin D (25(OH) Vitamin D) (a) and 1,25-hydroxy Vitamin D (1,25(OH)2 Vitamin D) (b) in IBD patients applying protein and carbohydrate- rich diet or balanced diet.

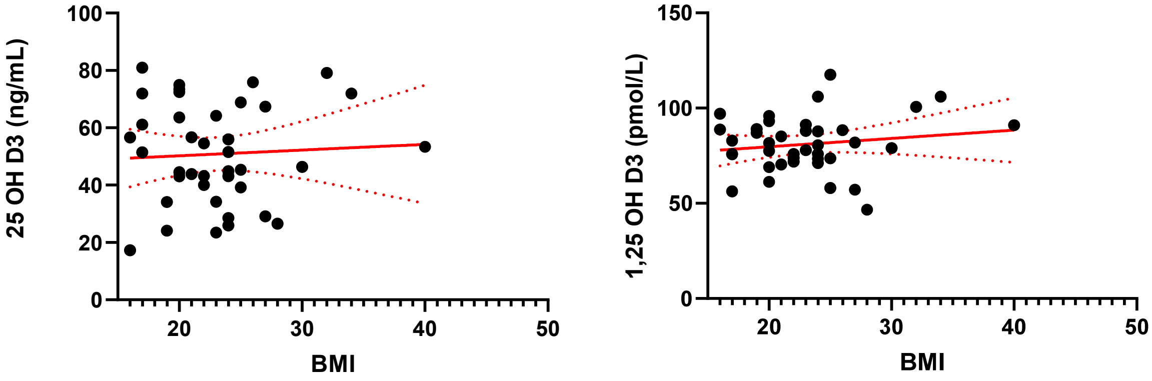

(**a**) (**b**)

Figure S2. Correlation between serum concentration of 25-hydroxy Vitamin D (25(OH) Vitamin D) (a) and 1,25-hydroxy Vitamin D (1,25(OH)2 Vitamin D) (b) and the values of body mass index (BMI).

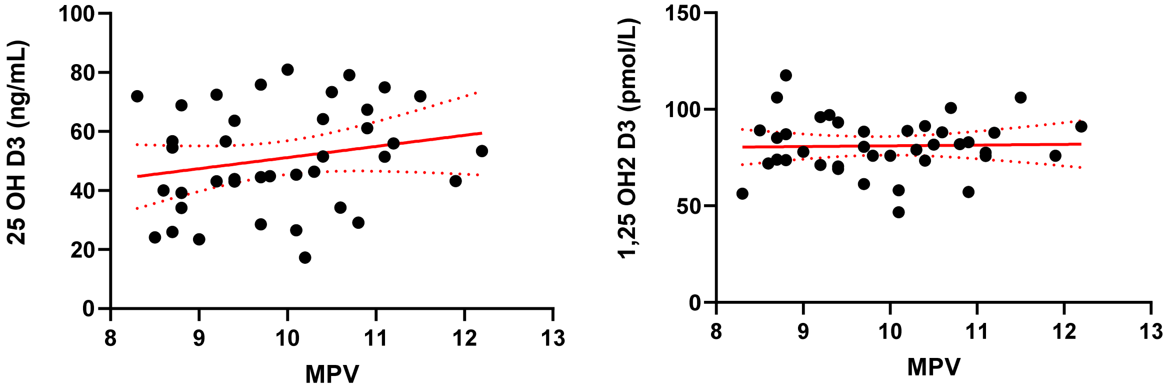

(**a**) (**b**)

Figure S3. Correlation between serum concentration of 25-hydroxy Vitamin D (25(OH) Vitamin D) (a) and 1,25-hydroxy Vitamin D (1,25(OH)2 Vitamin D) (b) and the values of mean platelet volume (MPV) in IBD patients.

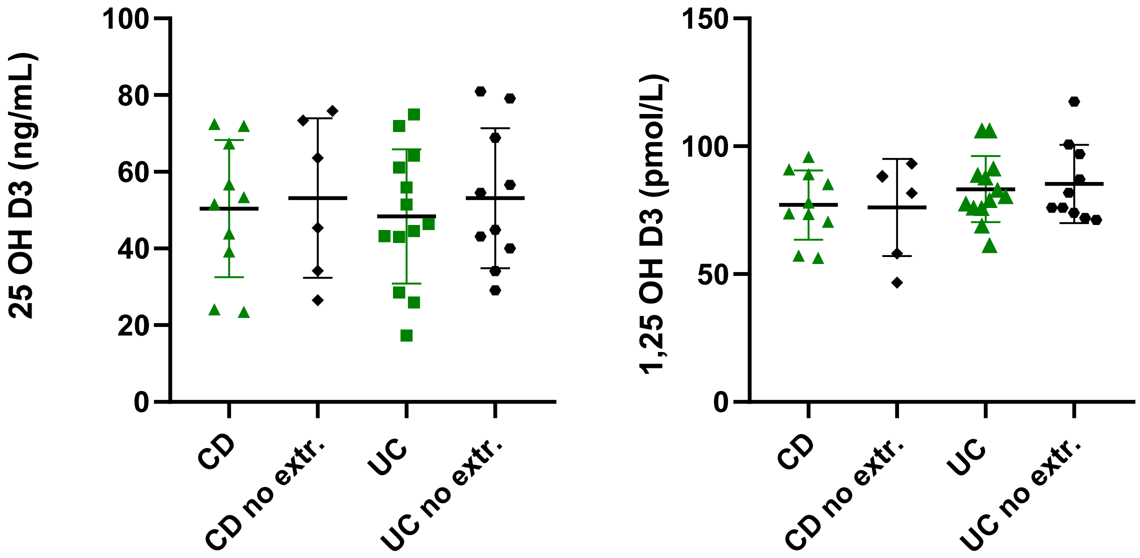

(**a**) (**b**)

Figure S4. Serum concentration of 25-hydroxy Vitamin D (25(OH) Vitamin D) (a) and 1,25-hydroxy Vitamin D (1,25(OH)2 Vitamin D) (b) in IBD patients with and without extraintestinal symptoms.
